# The effects of providing peer feedback on learners’ genre awareness in English as a foreign language business letter writing

**DOI:** 10.3389/fpsyg.2022.1059555

**Published:** 2022-12-01

**Authors:** Huizhen Wu, Huimin Zhu, Xiaohu Yang

**Affiliations:** ^1^School of Foreign Languages, Tongji University, Shanghai, China; ^2^Faculty of Foreign Languages, Shanghai Business School, Shanghai, China

**Keywords:** peer feedback, business letter writing, generic features, audience awareness, effective communication, English as a foreign language writing

## Abstract

Despite the important role of the genre awareness in facilitating the effective communication in the global business context and the need to teach and practice writing for various social purposes, there was scant classroom-based research on effects of providing peer feedback on EFL learners’ genre awareness in business writing for fulfilling the particular communicative purpose. This study examined whether student reviewers could improve their genre awareness in business letter writing by providing peer feedback to other students’ writings at varying levels. Sixty business English majors, taking the business writing course for 1 year under the tutelage of the same instructor, participated in the current study. They were randomly assigned to the experimental group who reviewed the students’ drafts at different levels (high, medium and low) and gave written comments and the control group who received no treatment but did self-revision. Both groups followed the genre-specific evaluation criteria during peer feedback and self-revision. Repeated-measures ANOVA on the two groups’ writing performances at different timepoints (pretest, immediate posttest, transfer posttest and delayed transfer posttest) demonstrated that the participants in the experimental group had better performance than those in the control group at the transfer posttest as well as the delayed transfer posttest. Moreover, providing weakness comments on both the low-quality and the medium-quality drafts had significant effects on their own writing quality at the subsequent tests, whereas providing strength feedback on the high-quality drafts had statistically significant impacts on their own writing quality at the immediate posttest and the delayed transfer posttest. However, multiple linear regression analyses demonstrated that only offering weakness comments to the medium-quality drafts could effectively predict the reviewers’ overall writing quality in the immediate posttest, the transfer posttest, and the delayed transfer posttest. Tentative research and pedagogical implications of the findings were discussed.

## Introduction

*Right now it frustrates me that I cannot write them [business letters] faster, but I have to remember that I’m new at this. One of my problems is patience—I expect to jump into a new job and know all about it and what to do right away. Realistically, this is impossible. It takes time to learn these things—especially when I’m on my own.*

This excerpt is from a 21-year-old student (Anson and Forsberg, 1990, p. 214) undergoing difficulty writing in the context of a student internship at a large company. It showcases the transitions that writers make when they shift from an academic to a nonacademic setting and embark on writing in a new and unfamiliar professional setting. As Schneider and Andre (2005) put it, tacit learning of genres can and will occur once students are immersed in the workplace, but such learning—typically through trial and error—can be slow and frustrating. Business writing does differ significantly from academic writing insofar as business organizations differ from academic organizations, because the writing within these organizations serves different purposes, addresses different audiences, and arises in response to a very different set of problems^1^.

Universities play a very important role in preparing students for workplace writing although the academic and workplace writings are “worlds apart” (Dias et al., 2013) and the transfer of writing skills from the classroom to the workplace is a complex phenomenon (Schneider and Andre, 2005). Business language instructors in departments of modern languages must recognize that the writing learners will be required to do on the job is different from most academic writing learners are practicing in humanities classes (Vásquez, 2013, p. 99). Fortunately, curriculum designers have begun to realize the need to teach and practice writing for various social purposes (Yu et al., 2022). Undoubtedly, being able to analyze, write and/or translate business or professional genres has become an essential skill for L2 learners and in-service employees in multicultural and multilingual megapolis or city clusters (Wen et al., 2022, p. 1). As such, business communication and trade transactions are growing exponentially, conducted predominantly in English, which has been propelled by the corporate *Englishization* process in more and more multinational corporations (Goldkorn et al., 2022). As put in Perkins and Salomon’s (1992, p. 3) words, “the ends of education are not achieved unless transfer occurs.” Therefore, writing taught in universities as a social practice should go beyond the purpose of merely learning writing skills and students should be situated in authentic or simulated writing contexts to be prepared for the multiplicity of writing demands they will face in the workplace (Yu et al., 2022).

Despite the necessity of preparing students for the workplace writing, the current Chinese EFL writing curriculum has been mainly confined to classroom contexts, the absence of social practice discourse with authentic social purposes in the EFL writing syllabi seems to indicate that the social potential of writing for authentic purposes and for making social changes has yet to be fully tapped (Yu et al., 2022). At undergraduate levels, EFL writing instructors often assign topics that incorporate rhetorical devices and ask students to construct argumentative essays, many of which share a similar structure (introduction with thesis, body, and conclusion). In contrast, business writing is primarily transactional in nature by seeking to inform and often recommend a course of action, and business writing in each of its genres has its own structure and often a somewhat rigid format to be followed closely (Vásquez, 2013). In the inquiry into the question whether learning standard “school” genres such as the academic essay or research paper translate into useful knowledge in the world of writing white papers, newsletters, proposals, laboratory reports, and business letters, Beaufort (1998) proposed that teachers could do a lot to shape curriculum that prepares students for the multiplicity of writing demands they will face in the workplace. To borrow what Moore and MacArthur (2012) put, whatever tasks or genres, adolescents deserve carefully designed instruction to prepare them to write clearly and logically for a range of purposes and audiences so that they can be successful students, workers, and citizens of a democratic society.

Writers must consider the context of their writing and for whom their writing is intended and the ability to consider one’s audience when forming an utterance marks a milestone in cognitive and linguistic development (Traxler and Gernsbacher, 1993; Holliway and McCutchen, 2004). Business letter writing, in particular, entails audience awareness because the writer must consider the perspectives of an audience to achieve the communicative goal of conveying information to the audience with clarity and purpose. Peer feedback as an instructional practice has a significant positive overall effect on students’ writing and provides writers an opportunity to find out how well their writing communicates to readers and to learn the writing of their peers by reading and evaluating (Moore and MacArthur, 2012; Vuogan and Li, 2022). However, this positive effect of peer feedback on business letter writing remains underexplored. With a view to connecting the simulated writing in EFL classroom with the authentic practical writing in the professional world, this study attempted to address the effectiveness of raising learners’ genre awareness by utilizing the peer feedback practice in teaching practical writing, i.e., business letter writing, in order to better prepare learners for future professional careers in the targeted discourse community.

## Literature review

### A genre approach to business letters writing

A formal business letter is a type of communication between a company and an individual or between individuals and companies, such as contactors, clients, customers, and other outside parties. Business letters, though frequently either sent by fax or replaced by fax messages in this digital age, remain an important genre of communication in business setting and are still very often the main means of establishing business relations with other organizations (Taylor, 2005; Bargiela-Chiappini et al., 2013). Furthermore, business letters represent a specific communication event in which there is a close match between the intentions of the writer and the expectations of the reader (Jenkins and Hinds, 1987). Wen et al. (2022) postulated that being able to analyze, write and/or translate business or professional genres has become an essential skill for L2 learners and in-service employees in multicultural and multilingual metropolises.

The term genre is defined as “a class of communicative events, the members of which share some set of communicative purposes” (Swales, 1990, p. 58). Genre approaches regard writing as predominantly linguistic but they emphasize that writing is tied closely to a social purpose and varies with the social context in which it is produced (Badger and White, 2000). It is most often a highly structured and more or less standardized communicative event with constraints on allowable contributions in terms of their intent, positioning, form and functional value on the part of the participants (Bhatia, 1991). Therefore, there emerge a variety of genres in business settings—such as shop transactions, sales promotion letters, service encounters—linked with specific situations (Flowerdew, 1993). However, the models of these genres are not fixed, rule-governed patterns, but rather “prototypes which are subject to individual variation” (Flowerdew, 1993, p. 308).

Explicit instruction in typical genre features simply provides a foundation or a schema upon which students can build further as they refine their local understandings of genre production within particular contexts. In Schneider and Andre’s (2005) investigation of how Canadian university student interns in three disciplines (Management, Communication studies, and Political science) perceived their educational preparation for workplace writing, they found students who expressed satisfaction with their preparation for workplace writing pointed to the familiarity they had developed with the particular genres they were called upon to produce in the workplace (Schneider and Andre, 2005). However, as shown in the excerpts in the Introduction, when interns struggled to master unfamiliar genres, they might experience a sense of “frustration and failure” (Anson and Forsberg, 1990, p. 214) which often originated from lack of knowledge about conventions of the writing context. Reflecting upon publications covering aspects of letter and report writing, which were among the earliest Business English materials, St John (1996) lamented that the materials only provided examples and models for learners to copy but did little to develop language awareness and the planning and organizing of data required by a reader-centered writing process were not emphasized.

Taking a Hallidayan framework (Flowerdew, 1993), there are three contextual parameters affecting genres: *field* (what the text is about), *tenor* (the relation between text producer and text recipient) and *mode* (the type and purpose of the text—written to be read, written to be spoken, etc.,). For example, sales promotion letters are in the *field* of a particular product or service, *tenor* is that of business to a selected group of individuals or companies, and *mode* of written to be read. Typically, a sales letter makes use of seven moves in the following sequence (Bhatia, 1991, p. 157): establishing credentials, introducing the offer (offering the product/service, essential detailing of the offer, indicating value of the offer), offering incentives, referring to enclosed documents, inviting further communication, using pressure tactics, ending politely. It should be noted that there can be a certain degree of flexibility in that these moves do not necessarily coincide with paragraphs; neither is it obligatory to use all of them and in the same sequence, all depending upon the situation to which the letter responds.

Additionally, acceptable moves and their rhetorical and linguistic realizations can be both “culturally-and language-bound” (St John, 1996, p. 7). Even second language learners from varying subcultures within the same cultural tradition convey politeness in their business letter writing differently. Qian and Pan (2019) in their purpose-built learner corpora examined how Hong Kong and Shanghai tertiary-level learners of English tracked a rather uneven and complex distribution of politeness realizations reflected in their different modal verb uses. Their findings revealed that Hong Kong ESL learners appeared to be more strategic users of modal sequences and were able to use more *might-related* modal sequences to soften the tone of writing and sound more objective. The study also revealed that the HK-U group has used significantly more expressions containing would-and could-related modal sequences, which would make the letters sound more diplomatic (Qian and Pan, 2019).

Learners are expected to have an informed understanding of how to write some typical genres (e.g., sales letters) in accordance with the established conventions shared by the targeted *discourse community*. Aside from some mastery of generic features, learners also need to understand the general conventions in the formats and structures like “layout of a typical formal business letter” (Wen et al., 2022) or termed as “an underlying constant of formality” (Hyland and Jiang, 2017), for example, where and how to put the sender’s address, the inside address, the date, salutation, the content, the complimentary close.

To sum up, despite the genre-based pedagogy advocated in the EFL business writing classroom, a reader-centered writing process aiming at realizing effective communication still holds a marginal place. Considering that research regarding how to effectively raise learners’ genre awareness in business writing remains underexplored, the present study attempts to address it from the perspective of student writers’ providing feedback on their peers’ business letter writing.

### Peer feedback as an instructional approach

Peer feedback is a reciprocal activity during which learners at similar or identical education levels provide and/or receive task-relevant feedback on their peers’ writing in the written, verbal or online mode in pairs or small groups (Zhu, 2001; Philippakos and MacArthur, 2016) and may contribute to language learning across multiple educational settings (Double et al., 2020; Li et al., 2020). It refers to “the use of learners as sources of information and interactants for each other in such a way that learners assume roles and responsibilities normally taken on by a formally trained teacher, tutor, or editor in commenting on and critiquing each other’s drafts in both written and oral formats in the process of writing” (Liu and Hansen, 2005, p. 1).

In order to raise writers’ reader awareness and place learners at the center-stage to enable them to have agency to critique their peers’ L2 output, Vuogan and Li (2022) proposed a tripartite framework encompassing three interrelated perspectives: peer feedback as information processing, peer feedback as interaction, and peer feedback as an instructional package. From the vantage of peer feedback as information processing, peer feedback interaction contributes to learning by prompting the *feedback provider* to scrutinize the peers’ compositions, apply their L2 knowledge to evaluate their quality, and formulate feedback to assist the writer. This inferencing process in reviewing cognitively engages the reviewers in perusing peers’ drafts and deducing the writer’s intended meaning so as to “provide feedback targeted at clarifying the intended message” (Vuogan and Li, 2022, p. 3). In the view of the peer feedback as interaction, peer feedback interaction can be more easily deciphered and is more relatable because learners have shared backgrounds and proficiency levels. This view also echoed Cho and MacArthur’s (2010) observation that student writers might understand peer comments more easily than expert comments because peers share problems, languages, and knowledge. Besides, peer feedback interaction may indirectly effect L2 writing and learning more broadly because of enhanced learner autonomy, self-efficacy, and motivation. Finally, peer feedback is operationalized as an instructional package, which comprises multiple components such as training, feedback, and revision, all likely to contribute to L2 writing ability (Vuogan and Li, 2022).

Another theoretical perspective supporting peer feedback is *learning-by-reviewing* hypothesis postulated by Cho and MacArthur (2011). From this perspective, reviewing actively involves reviewers not only in understanding one another’s drafts of different quality but also in participating in an evaluative process of detecting, diagnosing problems as well as generating solutions to address the problems. This involvement as a motivational-cognitive construct, according to Laufer and Hulstijn (2001), is contingent upon three components: need, search, and evaluate. The *need* component, as the motivational dimension of involvement, is the need to achieve in its positive sense based on a drive to comply with the externally imposed of self-imposed task requirements. Search and evaluation are the two cognitive information-processing dimensions of involvement, contingent upon noticing and deliberately allocating information to certain tasks. Therefore, this *learning-by-reviewing* approach naturally provides writers with audience awareness perspective in order to better convey the intended message. The current study was grounded in these two theoretical perspectives to explore the effect of applying peer feedback in business writing context and to delve into the effectiveness of providing different feedback comments for peers’ drafts of varying levels in reviewers’ own writing performance.

Quantitative research examining the effects of peer feedback as a frequent feature of second language (L2) writing instruction has gained momentum since 2010 and proliferated in the past two decades (Vuogan and Li, 2022, p. 10). Advocates of peer feedback in extant studies contend that its potential benefits have spanned from fostering learner autonomy (Hyland, 2000), supplementing rather than supplanting teacher feedback (Lee, 2009) and AWE feedback (Yao et al., 2021), benefits from receiving as well as giving feedback (Rouhi et al., 2020), nurturing a broad spectrum of perspectives and building rapport among students (Rollinson, 2005), raising audience awareness (Tsui and Ng, 2000), to facilitating a convergence of input and output by exposure to positive and negative evidence during reading and reviewing L2 texts (Vuogan and Li, 2022). A recent meta-analysis confirmed that compared with students who do not participate in peer assessment, those who participate in peer assessment show a significant increase in their writing performance (Li et al., 2020).

However, the utility and efficacy of peer feedback remains controversial. One of the concerns results from L2 learners’ distrust in the accuracy of feedback from peers with a similar proficiency level (Tsui and Ng, 2000; Hyland and Hyland, 2006; Yang et al., 2006), for example, the learners’ negative affective engagement due to their tendency to trust their teachers as reliable sources of grammar knowledge (Saeli and Cheng, 2021).

Other studies delved into the different benefits brought by peer feedback and self-revision in that the former advantages topic development while the latter elevates lexicon (Suzuki, 2008). Relatively fewer empirical studies addressed the effects of learners’ providing peer feedback on the feedback-givers’ potential learning gains (Lundstrom and Baker, 2009; Cho and Cho, 2011; Cho and MacArthur, 2011; Patchan and Schunn, 2015). To our knowledge, Cho and MacArthur’s (2011) study was among the fewest empirical studies exposing student reviewers to multiple peer drafts of low-, medium-, and high-quality to examine if the students would learn about writing from the experience of rating and commenting on papers written by their peers. Learners in the experiment were required to write on the introductory section of a lab report. They proposed that the *learning-by-reviewing* approach might provide students writers’ viewpoints or audience knowledge and argued that the problem-diagnosis experience might help reviewers understand the sources of the problem and inform them how to repair the problem. They found that only problem detection of the weakness comments best predicted the reviewers’ writing quality. However, the effect of comment scopes regarding which type of the multiple peer drafts (of low-, medium-, and high-quality) influences the writing quality remains unexplored. Cho and Cho (2011) demonstrated that providing weakness comments for micro-meaning and strength comments for macro-meaning improved the reviewers’ writing qualities and that the quality of reviewed peer drafts influenced the types of comments given in the lab report writing. In Patchan and Schunn’s (2015) research, a significant interaction of reviewer ability and text quality was reported, with high reviewers describing more problems in the low-quality texts than in the high-quality texts. Their findings demonstrated that the quality of the paper being reviewed was expected to affect how much practice is available to a reviewer, that is, low-quality texts presumably have more problems than high-quality texts and thus provide more opportunities for problem detection, diagnosis, and selection of appropriate solutions. However no significant effects of text quality were found. The question about whether low-quality texts can offer more opportunities to practice revision skills than high-quality texts is unanswered.

Perkins and Salomon’s (1992, p. 8) proposed two distinct but related mechanisms for transfer: the *low road* transfer, which depends on extensive practice and automaticity, and *high road* transfer, which depends on mindful abstraction from the context of learning or application and a deliberate search for connections. In a similar vein, Nelson and Shunn (2009, p. 376) further made a distinction between *performance* and *learning* for considering the effects of feedback in writing. *Learning* is the knowledge gain observed on transfer tasks, e.g., new writing assignments while *performance* is the knowledge gain observed on repeated tasks, e.g., multiple drafts of the same writing assignment.

Philippakos and MacArthur (2016) examined the effects of giving feedback on the quality of the reviewers’ own persuasive writing by subjecting fourth-and fifth-grade 145 students to reviewer, reader control, and time control conditions. The findings revealed that students in the reviewer group included more elements to address the opposing position and end with a message to the reader on the immediate posttests, and reviewers produced better quality final essays than both control groups did on one immediate posttest and the transfer posttest, and better essays than the reader control group did on the delayed transfer.

Based on the literature on genre-based approach to business writing and existing findings on peer feedback in the EFL writing context, the following aspects warrant further exploration. First, previous studies focused mainly on the peer feedback in genres like persuasive, narrative writing, while the practical writing genre remains marginalized and underexplored despite its utility in the students’ future career. Second, few studies attended to the comment scope on reviewers’ writing performances. As depth of processing and the degree of involvement may differ across tasks, it will be more revealing to tease out the effects of providing different commenting styles relating to the draft quality on reviewers’ writing performances across different tasks. Third, regarding the selection of peers’ drafts, most previous research tended to opt for the high-quality and/or low-quality texts for learners to provide feedback to examine the learning outcomes. What remains underexplored is the effectiveness of providing peer feedback for the medium-quality drafts, which most of our students may produce in an EFL writing context. Finally, most studies tended to use the overall writing quality at the posttest as the learning outcomes, and we argue that it would also be meaningful to gage if the peer feedback practice can have any impact on learners’ genre awareness measured at different phases to see its sustained effect in the business letter writing context.

### The current study

Learners need to understand the generic features as well as the “underlying constant” of business letters in order to achieve effective communication (Hyland and Jiang, 2017). However, since reader-centered writing process is often not emphasized in business letter materials, materials provided for business letter writing could do little to help learners develop language awareness (St John, 1996). As pointed out by Cho and MacArthur (2011), peer feedback could lead to improved writing by enhancing writers’ understanding of the perspectives and needs of readers. It helps induce different degrees of involvement load *via* searching and evaluation, and may enable student reviewers to allocate attention to the conventions and generic features of business letters. But the role that comments offered to peers’ writings by feedback providers (student reviewers) in their own writing outcomes remains unclear. This study was designed to explore the effectiveness of providing different types of comments for drafts of varying quality on feedback providers’ business letter writing at different timepoints: the immediate posttest, transfer posttest and delayed transfer posttest. Specifically, this study seeks to answer the following research questions:

RQ1 Does providing peer feedback exert any effects on learners’ learning outcomes in business letter writing?
RQ2 Does the type of feedback comments (weakness and strength) contribute differently to providers’ learning outcomes?
RQ3 Does reviewing students’ writings at varying levels (low-, medium-, and high-)exert any differential effects on improving feedback providers’ genre awareness in business letter writing?

## Materials and methods

### Participants

This study was conducted in the Faculty of Foreign Languages at a public university in eastern China. All participants were second-year Business English majors and most of them would be prepared to work for international corporations or institutions, where English is necessarily a working language in business communication. They were randomly assigned to an experimental group (*n* = 30) and a control group (*n* = 30). Table 1 gives detailed information about learners’ demographics and English proficiency. They were all registered in a 1-year syllabus-based business English writing course, and attended a 90-min class once a week under the tutelage of one instructor. Analyzes were conducted to check for differences by group based on the latest scores of the standardized College English Test Band 4 (CET-4) that the students took in the winter of 2021 and the final grades of the business English writing course in the fall semester of 2021(i.e., last semester) respectively. The national CET-4 is a comprehensive written English test to examine university students’ listening, reading, and writing proficiency (Zheng and Cheng, 2008). Independent samples t-tests showed no statistically significant differences between the groups regarding the age (*t* (58) = 0.867, *p* = 0.39), the CET-4 scores (*t* (58) = −0.605, *p* = 0.547) and the writing performance (*t* (58) = 0.550, *p* = 0.585). Chi-square test confirmed that distribution for gender did not differ (*χ^2^* (2, Number = 60) = 0.577, *p* = 0.448) between the two groups.

**Table 1:** Participants’ information.

Conditions	Number	Age *M* (SD)	CET-4 (2021) *M* (SD)	Writing scores (2021) *M* (SD)
Experiment	30 (*F* = 25, *M* = 5)	19.8 (0.83)	558.57 (48.42)	75.17 (8.16)
Control	30 (*F* = 27, *M* = 3)	20 (0.64)	551.13 (46.68)	76.40 (9.19)

### Materials

Students in the experimental and control groups were given the same prompts at the pretest, immediate posttest, transfer posttest, and delayed posttest to write business letters on different topics (see Appendix A). The difference between the two groups was the different materials assigned for them to review. The experimental group reviewed the peers’ drafts at different levels whereas the control group did self-revision.

The experimental group were given six example drafts at three levels (high, medium, and low), with two drafts representing each level. These example drafts were selected from the control group at the pretest to represent a range of quality based on ratings by the researchers and the course instructor (see Appendix B for example papers). The selected six writings ranged in the overall quality from low, medium to high, by taking into consideration the layout, content, style and tone, and accuracy as per the scoring rubrics (see Appendix C for descriptors of each component) developed by the London Chamber of Commerce and Industry (LCCI) (Sharples, 2001). The control group were asked to review their own drafts produced at the pretest based on the rubric.

### Procedures

**Figure 1:**
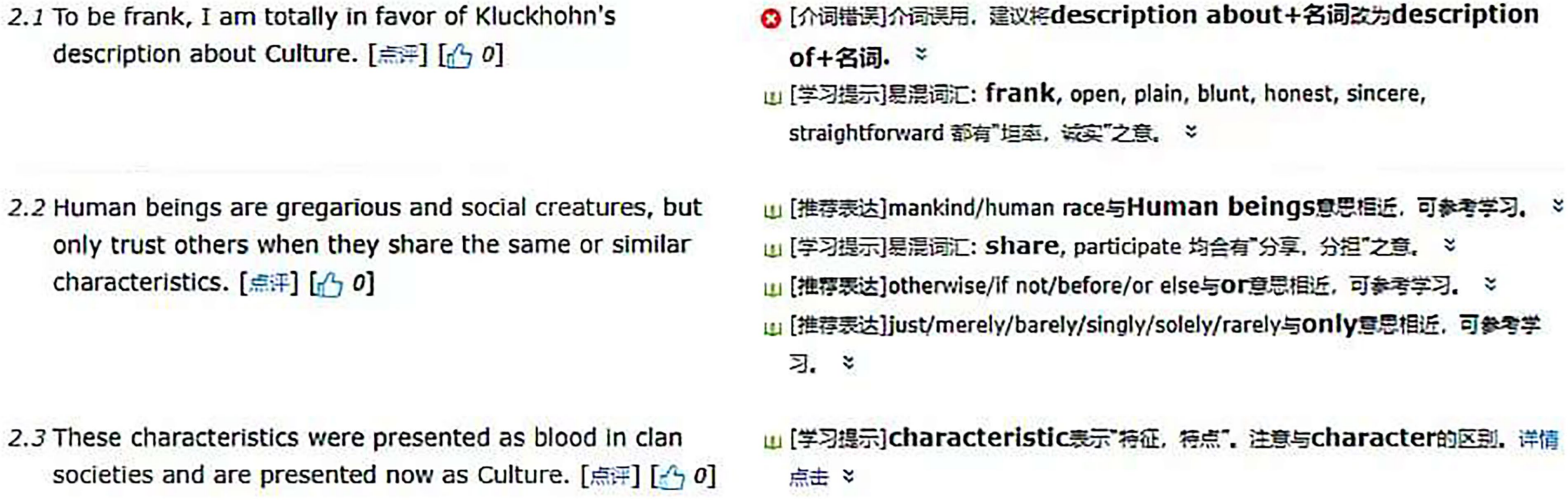
A screenshot of *Pigai* program.

The study was conducted over 8 weeks (see Table 2). Usually, the instructor conducted a group discussion of the formal features of the targeted business letter genre for all students. According to (Freedman, 1993), explicit discussions of the formal features of genre may prove useful when such discussions are presented while students are engaged in authentic reading and writing tasks involving the targeted genre. Participants of the present study normally received evaluation of their work from both the teacher and an AWE program^2^ in the writing instruction. As *Pigai* provides holistic ratings and detailed comments based on grammar, vocabulary, syntax, organization, and due to its accessibility and convenience, this program has been widely adopted in English writing instruction at the tertiary level in China (Jiang et al., 2020; Jiang and Yu, 2022). Figure 1 is a screenshot of the feedback provided by *Pigai* program. This program can enable multiple iterations of feedback, such that students can address mechanical errors and basic organizational or structural issues before submitting to their instructor. This would enable instructors to dedicate their energy toward feedback on content (Link et al., 2020; McCarthy et al., 2022).

**Table 2:** Procedures of the experiment.

Week	Experimental group	Control group	Writing prompts
1	Pretest (40 min)	Pretest (40 min)	See Appendix A for details
2	Peer feedback training	Self-revision training	
3, 4	Intervention sessions: peer feedback on anonymized drafts of different quality	Self-revision: revision of learners’ own drafts	
5	Immediate posttest (20 min)	Immediate posttest (20 min)	
6	Transfer posttest (40 min)	Transfer posttest (40 min)	
8	Delayed transfer posttest (40 min)	Delayed transfer posttest (40 min)	

In week 1 (Pretest), participants were required to write within 40 min the first drafts in response to a scenario—*an invitation letter*. To minimize the effects of language proficiency on feedback providers’ evaluation of learners’ genre awareness, participants’ first drafts were required to submit to the AWE *pigai* program to receive the automated feedback, according to which all participants revised the first drafts and then submitted their second drafts to the course instructor *via* email. Performance at this pretest stage provided a baseline against which performance in the subsequent three tests could be compared because, at the pretest stage, none of the writers had received any form of peer, instructor, or self-feedback.

In week 2, all participants received evaluation training using genre-specific rubric pertaining to the following elements: layout, content, style and tone, and accuracy in order to achieve the optimal communication with the recipient. Only the experimental group received peer feedback training to get familiar with the procedures in the first 2 weeks. It has been found that the most critical factor likely to influence the peer assessment effect is rater training. According to the study of Li et al. (2020), when students receive rater training, the effect of peer assessment is substantially larger than when students do not receive such training. The training included six elements: awareness-raising, explanation of procedures demonstration, practice, reflection, and instruction. Specifically, the students were asked to read 2 writings they composed during the previous semester, assigned a score for each element on a 5-point Likert scale, and provided written comments, including strength and weakness, in order to emulate and make further improvements. Students were reminded that reviewing means offering honest, courteous, and constructive feedback to the writers to help another writer to improve their work.

In week 3, the experimental group participated in peer review activities, during which they accessed *via* a link to read, rate and review 3 anonymized writings in a random sequence. Specifically, students were instructed to follow the evaluation rubric to identify strengths, detect weaknesses, describe problems, and generate solutions regarding each of the peers’ drafts. To minimize the effects of students’ language use on generating comments, all students were required to produce their written comments in students’ L1, Mandarin. Each student worked independently to evaluate the drafts in the 90-min class and were allowed to continue reviewing after class if time was inadequate for them to complete reviewing during class.

In week 4, the same procedure was repeated for evaluating another 3 anonymized writings.

The control group in weeks 3 and 4 were asked to review their own work based on the rubric. They accessed a link to note down the strength and weakness in their own writings and typed whatever changes they have made during revision. For both groups during weeks 3–4, the course instructor would assist them during revision if they sought help from her.

In week 5 (Immediate posttest), all participants were given 20 min to revise their drafts on an invitation letter during class and submitted them to the course instructor *via* email.

In week 6 (Transfer posttest), in order to assess the transfer effect, the participants were given a time limit of 40 min to respond to a new scenario—*looking for a new market* and submitted to the instructor *via* email.

In week 8 (Delayed transfer posttest), all participants were given 40 min to write a business letter in response to a different scenario—*invitation for an interview* and submitted to the instructor *via* email during class.

The experiment was carried out during regular course instruction. There were no missing data throughout the stages at pretests, immediate posttests, transfer posttests, and delayed tests. Participation in the study was voluntary and was considered in the formative assessment of the business English writing course. Students’ oral and written consent were obtained from all participants before conducting the experiment.

### Data collection

#### Writing quality

Following the rubric developed by the LCCI, two senior instructors of writing independently graded the anonymized 240 drafts (30 × 2 × 4) to understand more fine-grained dimensions of essay quality by giving sub-scores of each component, including layout (heading, inside address, salutation, body, closing, and signature), content (key points, additional details and additional invented details), style and tone (writer and addressee relationship, purpose), and accuracy (spelling, grammar, and punctuation). Participants’ personal information was anonymized and the two raters were also blind to the experiment condition. The possible maximum total score is 30 points. Both raters received about 2 h training on the scoring scale. Training included first explanation of the rubric, then practice by observing the rubric with three example papers, and finally discussion. The interrater agreement reached 94.7, 96.7, 96.2, and 95.7% for layout, content, style and tone, and accuracy, respectively. Finally, the authors discussed with the raters to address all the disagreements regarding the two raters’ scoring.

#### Coding scheme for peer review comments

The coding scheme (Table 3) for comments’ type and value was adapted from the models developed by Patchan and Schunn (2015) and Novakovich (2016). The comments include both strength and weakness. A comment under the strength category was coded as “improper,” “naïve” or “pertinent” comment unit. A comment under the weakness category was coded as “improper,” “naïve,” “editing,” “critical” or “directive” comment unit. Previous research has indicated that students might have difficulties identifying problem areas in other students’ writing and offer them inaccurate or misleading advice (Hyland and Hyland, 2006, p. 7). Considering this in operationalizing coding, we incorporated “improper” category to define the inaccurate or misleading advice and comments in our coding scheme. All ideas based on reviewers’ feedback (strength and weakness) are segmented into smaller comment units. Each idea unit was categorized and weighted based on the coding scheme. The comments generated by the participants in the experimental group were analyzed independently by two senior EFL teachers, who received about 2.5 h training on the coding scheme by the first author. Training included explanation of the scheme, then practice by first segmenting comment units and then using the coding scheme to code 10% of randomly selected written comments, and finally discussion. We examined the agreement on idea unit segmentation and comment categorizations and found that for the comment segmentation and categorization, the coders agreement initially reached 96.7 and 94.6%, respectively. All the disagreements were resolved *via* discussions. Altogether, the total number of comments, together with the value for each reviewer’s total comments and each subcategory was calculated.

**Table 3:** Coding scheme of peer review comments.

Category (value)	Definition	Example
**Strength**		
Improper (0 point)	Giving wrong comments	A very poorly written piece of work was commented as: *The draft is concise and easy to understand*!
Naïve (1 point)	Giving simplistic statements to show agreement	A very well written piece of work was commented as *Overall, the content of the article is easy to understand*.
Pertinent (2 point)	Giving proper comments regarding the layout, content, and tone of the draft	*The second paragraph focuses on expressing the advantages of its own products and the benefits it can bring to partners in the future. This, I think, is the highlight of this letter, because the advantages of the products are conveyed to the recipient at once, and the invitation letter can lay a solid foundation for follow-up cooperation*.
**Weakness**		
Improper (0 point)	wrong comments on the weakness of a draft	*The salutation should be “Dear Mr Roland”*
Naïve (1 point)	simplistic statements of opinion that are too general to be helpful or to lead to useful revision strategies	*The content of the games is not that exhaustive*.
Editing (2 point)	mechanical details pertaining to sentence structure, grammar, and punctuation, micro solutions or surface-level errors	*“to acquainted with each other” should be “to acquaint with each other,” “our new game is designed for” should be “our new games are…,” “will be productiv” should be “will be productive”*
Critical (3 point)	statements that evaluate the work and explain its effect on the reader	*The biggest problem of the problem lies in wrong interpretation of the situation. It’s clearly stated in the situation that the company will hold a game exhibition, and hope that the recipient will be present in order to help launch the newly released products*.
Directive (4 point)	ways to improve the work, reorganize the structure or ask questions to the author that would lead to further knowledge building and reflexivity	*The author should state clearly in the very first paragraph the time, place and date of the conference, and then detail in the next paragraph the activities to be held in chronological order*.

#### Data analysis

We first performed a repeated-measures ANOVA in SPSS to explore the changes in their writing qualities between the two groups across the experiment period. We also examined the correlations between comment types (weakness and strengths) and the writing qualities (low, medium, and high levels) measured by elements of layout, content, style and tone and accuracy in the experimental group. Associations among providers’ feedback types and their overall writing quality at different phases were examined with Pearson’s *r* for bivariate correlation. Then multiple linear regression analyzes (stepwise method) were conducted to detect the significant predictors (when all factors were taken into consideration) of specific feedback types on the elements of the students’ business letter writing across the study period. An *α* level of 0.05 was used for all statistical analyzes.

## Results

The results were addressed in 3 steps. First, the mean scores of the participants’ writings before and after the intervention were reported and compared across the group. Next, the associations between the participants’ comment types in the experimental group and their writing quality were examined. Finally, the regression results for the relationships between the feedback types and the components of the business English correspondence were reported.

### Writing quality before and after the intervention

**Figure 2:**
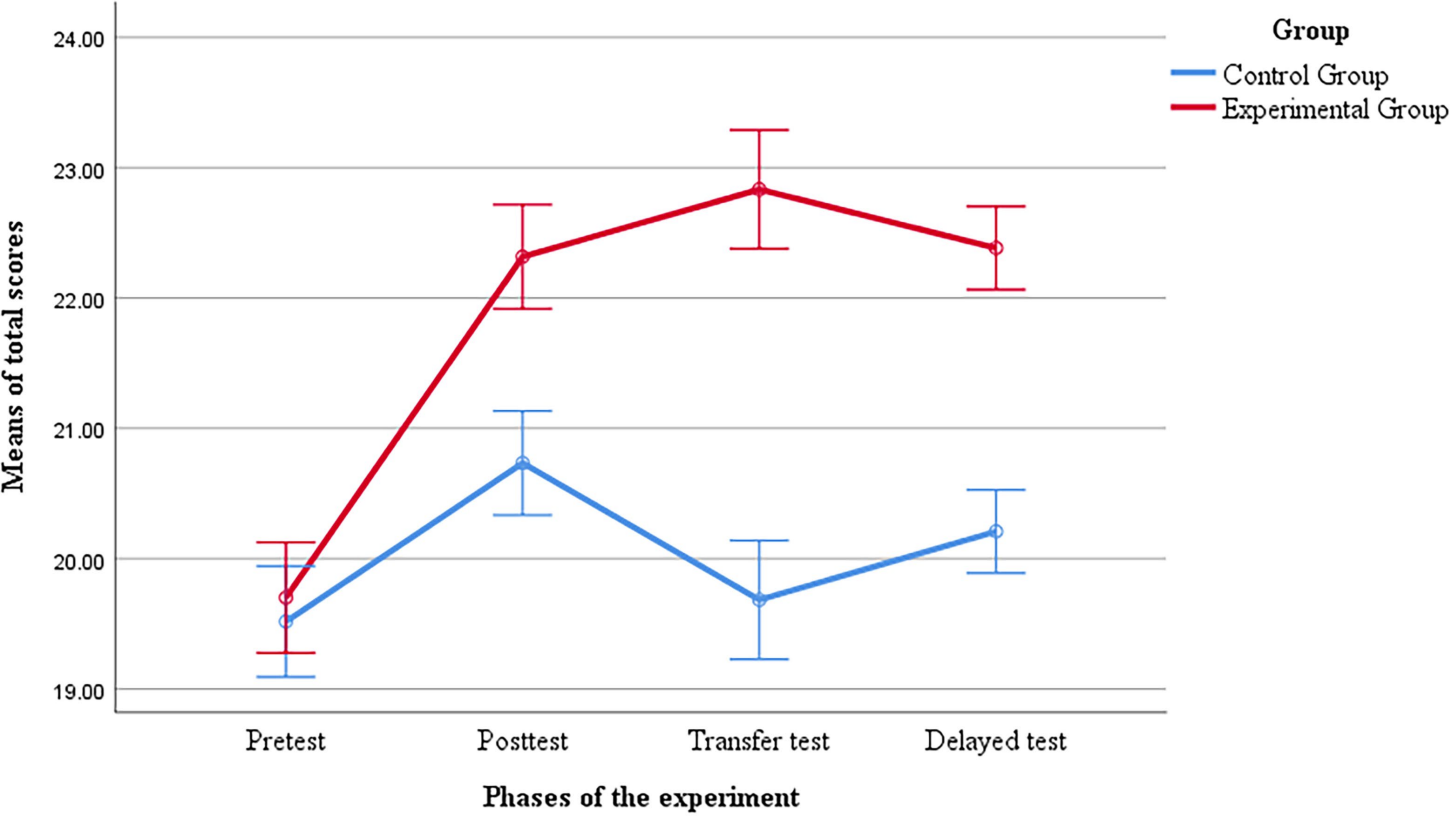
Writing qualities between the two groups at different phases during the experiment. The error bars enclose ± 0.5 *SE.*

In order to test whether there were significant differences between the two groups in writing performance over time, a repeated-measures ANOVA test was carried out. *Group* was treated as a between-subject variable (experimental group vs. control group) and time (pretest vs. immediate posttest vs. transfer posttest vs. delayed transfer posttest) as a within-subjects variable. Dependent variable was the total scores of the combined elements of the drafts: layout, content, style and tone, accuracy. The results indicated that *group* had no main effect (*F* (1, 58) = 3.213, *p* = 0.078) and *time* had a main effect (*F* (2.062, 119.577) = 7.336, *p* = 0.001). Moreover, the results showed a significant interaction effect between *time* and *group* (*F* (2.062, 119.577) = 3.37, *p* = 0.029, η_p_^2^ = 0.056, Greenhouse–Geisser correction). Pairwise comparisons with Bonferroni correction were performed to further investigate the changes across timepoints within each group. As displayed in Figure 2, the two groups’ mean scores of the drafts in the immediate posttest were very similar (*M* = 19.517, SD = 5.113 for the control group and *M* = 19.700, SD = 4.135 for the experiment group; *p* = 0.879), but they displayed different trajectories after the intervention. Non-statistically significance differences were observed at the time of the immediate posttest (*p* = 0.166). However, noticeable differences were found at both the transfer posttest and the delayed transfer posttest between the two groups, suggesting that peer feedback intervention enabled the experimental group to achieve higher gains in business letter writing compared with the control group.

It was noteworthy that the writing performance at the posttests, transfer posttests, and delayed transfer posttests for both groups improved in varying degrees compared with their performance at the pretests. For the control group, statistically significant differences only appeared from the pretest to the immediate posttest (*p* = 0.001). Such differences did not occur neither from the immediate posttest to the transfer posttest (*p* > 0.05) nor from the pretest to the delayed transfer posttest (*p* > 0.05). In contrast, discernable improvement in the experimental group was observed across the tests (pretest vs. immediate posttest, *p* < 0.001; pretest vs. transfer posttest, *p* < 0.001) and the effect was sustained in the delayed transfer posttest (pretest vs. delayed transfer posttest, *p* = 0.004). Specifically, relative to the writing quality at the pretest (*M* = 19.700, SD = 4.135), their performance improved noticeably at the posttest (*M* = 22.317, SD = 3.546), and this growth momentum was maintained steadily at the transfer posttest (*M* = 22.833, SD = 4.964) as well as at the delayed transfer posttest (*M* = 22.3833, SD = 3.86169).

### Correlations between comment types and writing quality

**Figure 3:**
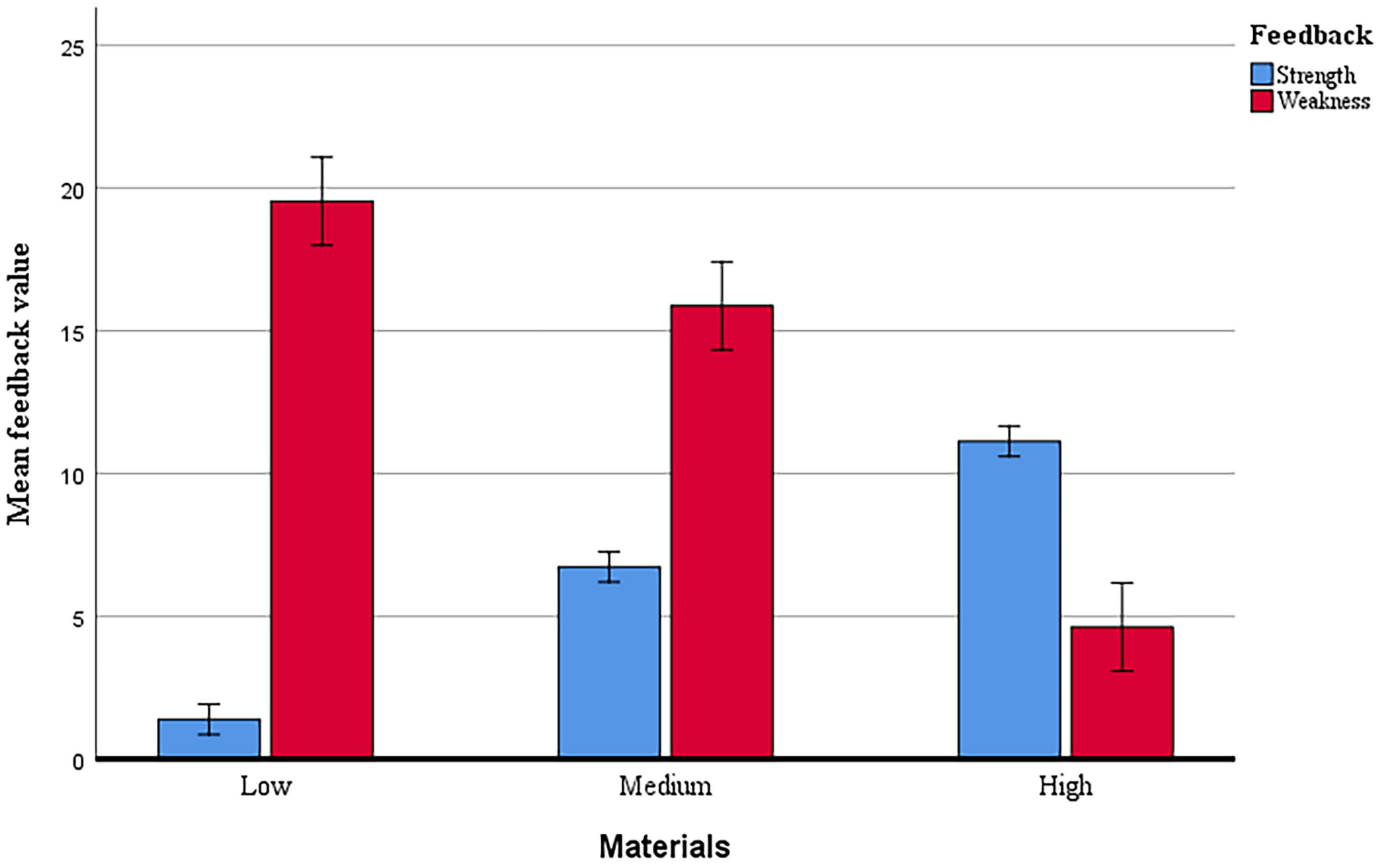
Comment types of low-, medium- and high-quality writings. The error bars enclose ± 1.0 *SE.*

Figure 3 displays the mean values of strength and weakness comments about the low-, medium-and high-quality writing. Table 4 shows the bivariate correlations among the providers’ feedback types of the low-, medium- and high-quality writings and their own writing quality at different phases after the peer feedback intervention, namely, the immediate posttest, the transfer posttest, and the delayed transfer posttest. Results showed that students’ providing strength feedback on the high-quality drafts had statistically significant correlations with their own writing quality at the posttests and the delayed transfer posttests (*r* = 0.364, 0.383 respectively; *p* < 0.05). Providing weakness feedback on both the low-quality and the medium-quality drafts had statistically significant and positive associations with reviewers’ own writing quality at the posttests, the transfer posttests, and the delayed transfer posttests (*r* = 0.468, 0.478, 0.495 for the low-quality drafts, and *r* = 0.696, 0.568, 0.540 for the medium-quality drafts; *p* < 0.01).

**Table 4:** Correlation matrix among feedback types and writing quality after the intervention.

	1	2	3	4	5	6	7	8	9
Strength-L	—								
Strength-M	0.305	—							
Strength-H	−0.035	0.568^**^	—						
Weakness-L	−0.266	0.292	0.756^**^	—					
Weakness-M	−0.263	0.355	0.687^**^	0.752^**^	—				
Weakness-H	−0.181	0.267	0.217	0.426^*^	0.367^*^	—			
TotalScore-2	−0.099	0.202	0.364^*^	0.468^**^	0.696^**^	0.106	—		
TotalScore-3	0.045	0.193	0.349	0.478^**^	0.568^**^	−0.122	0.733^**^	—	
TotalScore-4	−0.174	0.097	0.383^*^	0.495^**^	0.540^**^	−0.074	0.597^**^	0.756^**^	—

^*^p < 0.05 (2-tailed); ^**^p < 0.01 (2-tailed). L, low-quality drafts; M, medium-quality drafts; H, high-quality drafts; TotalScore-2, overall quality at the posttest; TotalScore-3, overall quality at the transfer posttest; TotalScore-4, overall quality at the delayed transfer posttest.

In terms of the weakness feedback type, the weakness comments on the medium-quality drafts bore significant relation to not only the participants’ overall writing quality in the immediate posttest, the transfer posttest, and the delayed transfer posttest. In comparison with the high-quality drafts, which boasted more strengths but fewer weakness, and the low-quality drafts, which have more weakness but fewer strength, the medium-quality drafts stood between the two extremes in terms of the quantity and the value of the strength and weakness. Among all types of feedback comments, only providing weakness comments for the low-and medium-quality drafts manifested significant correlations with all three tests. In other words, weakness comments for the drafts of low and medium quality had statistically significant positive associations with feedback providers’ own writing quality across all tests.

### Predictive effects of comment types on different elements of writing quality

To determine the unique contribution made by the feedback types to the specific elements of the writing quality at the posttest, transfer posttest and delayed transfer posttest, we then subjected the data to separate regression analyzes with layout, content, style and tone, and accuracy as dependent variables, respectively. Altogether 15 stepwise regressions were performed. Table 5 presents an overview of feedback types variable entered for each of the regression equations at different phases.

**Table 5:** Summary of stepwise regressions for predicting writing quality.

Dependent variables	Predictors	Adjusted *R*^2^	Standard Beta	*t*	Sig.
Posttest					
Layout	Weakness-M	0.164	0.440	2.58	0.015
Content	Weakness-M	0.340	0.602	3.991	0.000
Style and tone	Weakness-M	0.315	0.582	3.790	0.001
Accuracy	—	—	—	—	—
Total score	Weakness-M	0.466	0.696	5.13	0.000
Transfer posttest					
Layout	Weakness-M	0.194	0.471	2.827	0.009
Content	Weakness-M	0.255	0.530	3.303	0.003
Style and tone	Weakness-M	0.248	0.618	3.976	0.000
	Strength-L	0.349	0.359	2.308	0.029
Accuracy	—	—	—	—	—
Total score	Weakness-M	0.298	0.568	3.650	0.001
Delayed transfer posttest					
Layout	Weakness-M	0.157	0.432	2.533	0.017
Content	Weakness-M	0.133	0.404	2.336	0.027
Style and tone	Weakness-L	0.172	0.448	2.651	0.013
Accuracy	Weakness-L	0.207	0.484	2.925	0.007
Total score	Weakness-M	0.266	0.540	3.39	0.002

L, low-quality drafts; M, medium-quality drafts; H, high-quality drafts.

As shown in Table 5, the weakness comments provided for the medium-quality writings(weakness-M) contributed significantly to the total scores at all three phases after the intervention. This pattern was also captured in the layout and content performance.

Similarly, regarding the predictive effects of the feedback types on the specific elements of the overall writing quality, the weakness-M feedback consistently contributed the greatest amount of unique variance in the model attempting to predict the content scores at different phases in business correspondence writing. This particular feedback type accounted for 34, 25.5, and 13.3% of the variances at 3 phases, respectively, with positive beta weights (*β* = 0.602, 0.530, and 0.404).

Moreover, in assessing the variables influencing the style and tone scores at the 3 phases, the weakness-M feedback accounted for 31.5% of the variances (*β =* 0.582) at the posttest. At the transfer posttest, weakness-M and the strength-L were the two variables in the model, accounting for 24.8 and 34.9% of the variances (*β =* 0.618, 0.359 respectively). At the delayed transfer posttest, weakness-L accounted for 17.2% of the variance (*β =* 0.448).

For the relationship between feedback types and accuracy scores, only the weakness-L explained 20.7% of the variance at the delayed transfer posttest (*β =* 0.484). Furthermore, all three comment types (weakness-H, strength-H, and strength-M) failed to enter any of the regression equations to account for participants’ performance in business letter writing after the peer feedback intervention.

## Discussion

The current study addressed three questions: firstly, whether peer feedback activities would enhance reviewers’ genre awareness not only in the immediate posttest, but also in the transfer task and the delayed transfer posttest; secondly, whether different types of feedback comments could affect learners’ subsequent writing performances after the intervention in different ways; and finally, whether offering different types of comments to drafts of varying quality exerted differential effects on reviewers’ overall writing quality as well as their performances in the essential components of business letter writing. The answers to these questions were discussed in association with our findings from the experiment.

### Effects of peer feedback on reviewers’ learning outcomes in business writing

In answer to the first research question, the holistic scores for the experimental group and the control group at the pretest, immediate posttest, transfer posttest and delayed transfer posttest were assessed and analyzed. The results suggested that peer feedback did exert positive impact on enhancing learners’ learning outcomes in business letter writing. In other words, the two groups’ performances at the transfer and delayed transfer posttests differed noticeably because of the effectiveness of peer feedback intervention. The differences between the immediate posttest and the pretest for both groups did lend support to previous studies in that students who self-revise produce final draft of similar quality to students who provide peer-feedback (Suzuki, 2008). This could be justified because the control group in our study produced revised drafts at the immediate post. However, our findings revealed the effects of self-feedback failed to sustain during the transfer and delayed posttests. As suggested by Cho and Cho (2011), reviewing peer texts might be more helpful than reviewing one’s own texts because students had more difficulty detecting problems in their own texts than in their peers’ writing. Thus the uptake of the self-feedback practice for learners in the control group failed to be retained in the two novel tasks following the immediate test. In contrast, the peer feedback activities did produce effect in arousing learners’ genre awareness of the business letter writing and enabled the learners to perform equally well in different tasks.

Our finding echoed Nelson and Schunn’s (2009) argument that feedback affected learning and performance differently. Providing feedback without explanations may improve performance, but not learning. Learning gains occur in instances where students reflect on their own learning by providing feedback to others (Patchan et al., 2016). Only “deeper processing will lead to an improvement in memory” and hence contribute to learning (Craik and Lockhart, 1972, p. 681). This depth of processing can be explained by the involvement load construct, namely need, search and evaluate (Hulstijn and Laufer, 2001; Laufer and Hulstijn, 2001). In terms of giving feedback to peers’ writing, *search* is the attempt to find the strength and weakness in a peer’s text, whereas *evaluation* entails a comparison of the peer’s draft with the rubric and their own draft. Providing feedback on peers’ drafts of varying quality can induce all three components of involvement—*need*, *search* and *evaluate*. Processing for each task, however, may differ in the involvement load generated during providing feedback to drafts of different quality. Our findings echoed previous research that instruction in scoring rubrics coupled with practice in reviewing drafts of varying quality led to improvements in the quality of students’ own writing (Philippakos and MacArthur, 2016). Providing feedback to others especially with varying level of peer drafts entails a relatively greater number of cognitive efforts in noticing, critiquing and offering suggestions regarding the peers’ texts. This corroborates the *learning-by-reviewing* hypothesis (Cho and MacArthur, 2011) in that the reviewing process helps draw learners’ attention to the generic features of business letters by referring to the instructional packages. Pointing out the strengths of the peers’ drafts, together with explaining the problems and offering solutions seemed to help consolidate and internalize their understanding and acquiring of the generic features of business letters. Take the business letter writing tasks in our study as an example, communication of ideas with or without proper style and tone is of paramount importance, resulting in either success or failure of effective communication. The reviewing process put the student reviewers in readers’ shoes and when writers take the reader perspective, they are better able to understand what is needed for clear communication with an audience (Holliway and McCutchen, 2004). As writers usually do not have chance to receive immediate feedback while writing, they should predict the possible interpretations of their text so as to avoid misinterpretation. This audience perspective during writing helped writers avoid information gaps and improve “the communicative quality of their writing” (Holliway and McCutchen, 2004, p. 88).

### Correlations between comment types and reviewers’ writing outcomes

For the second research question regarding the correlations of comment types and feedback providers’ writing performances, our study indicated that the weakness comments seemed to be more effective than the strength comments in terms of the learners’ gains in their writing performance. Offering weakness comments may entail a series of more active cognitive processing than offering strength comments. In Hulstijn and Laufer’s words (2001, p. 544), the combination of factors (*need, search and evaluation*) with their different degrees of prominence constitutes involvement load. The two tasks, namely providing weakness and strength comments, might induce different degrees of involvement load. When providing weakness comments, learners first searched, detected, and then evaluated the problems on the reviewed drafts critically for offering explicit explanation and directive solutions according to their knowledge of the generic features. In contrast, strength comments involved only spotting the strengths on the drafts under review, requiring less time and attention in comparison with giving the weakness comments. This might lead to different degrees of involvement in the task, thus having differential effects on learners’ subsequent overall writing performances. The more involved learners were in reviewing, the more cognitive resources they would mobilize and the better performances they would score in their own writing. Guided by the scoring rubric during reviewing, students critically weighed different components of the business letters: layout, content, style and tone, and accuracy. Reviewing might sensitize feedback providers to notice the genre features and constituted a constructive learning activity in which student reviewers could internalize writing criteria and repair their ineffective writing strategies (Cho and Cho, 2011). Therefore, learners would generally benefit more from their cognitive engagement in giving weakness comments to the drafts with reference to the scoring rubric.

### Effects of reviewing different writings on learning outcomes

Our third research question inquired into the predictive effects of comment types offered to different levels of drafts on feedback providers’ writing performances. Our findings demonstrated that providing weakness comments about medium-quality drafts could best predict comments providers’ writing gains on the revised drafts, transfer task and delayed transfer task. As explained in the previous sections, offering weakness comments might be more cognitively demanding. However, critical comments and directive comments are key to high-road transfer (Reiff and Bawarshi, 2011). The participants in the experimental group were encouraged to detect problems, offer explanations, and give directive comments about the drafts under review. This might benefit reviewers and influence how they form and compose their own drafts. The medium-quality drafts seemed to be the optimal material for learners to provide feedback in the current study. One plausible explanation is that the degree of the cognitive involvement in providing comments for low-, medium-and high-quality drafts might differ. When reviewing high-quality drafts, learner’ attention might naturally befall the strengths of the drafts, with yet little room for improvement; when reviewing low-quality drafts, learners’ attention might be skewed towards the weakness of the drafts. Most of our learners produced medium-quality drafts, with fewer high-and low-quality drafts. They would find it far too difficult to detect deficiencies in the high-quality peer drafts. However, when reviewing low-quality drafts, as reported by Cho and Cho (2011, p. 632), undergraduate reviewers might learn little when explaining the strengths and weaknesses of surface features because they were likely to have adequate knowledge of spelling, grammar, and words. On closer examination, the weaknesses of the low-quality draft were dispersed in almost every element considered essential in a business letter, like neglect of conventions, miscommunication of ideas, improper style and tone, grammar, and misspellings. The comments were mostly confined to surface-level problems. Conceivably, compared with reviewing low-quality drafts, providing weakness comments about the medium-quality drafts would be more challenging to spot. This would encourage learners to draw upon their prior genre knowledge to understand the gap between what the text intended to convey and what they actually conveyed based on the written texts. Also, as suggested by Craik and Tulving (1975), what is crucial to retention is not simply the presence or absence of semantic encoding, but the richness with which the material is encoded. The reviewing process from reading, searching, interpreting, to evaluating peer drafts might involve student reviewers more deeply and enable them to develop a more accurate understanding of how readers would interpret their writing. The greater the involvement load, the better the retention (Hulstijn and Laufer, 2001). Therefore, the enhanced understanding of reader perspectives would help student reviewers to make matches between what they want to say and what readers would interpret and processing information more elaborately might lead to higher retention and better transfer effect than processing information less elaborately.

Our regression analyzes showed that feedback types did not exercise any significant influence on the learners’ performance in the accuracy element of the writing quality at the immediate posttest and the transfer posttest. We incorporated the use of *pigai* AWE program to make the learners’ writing achieve an acceptable level at the pretest stage in that it offers “holistic ratings and detailed comments based on vocabulary and syntax” (Jiang et al., 2020; Jiang and Yu, 2022). While AWE programs are able to identify errors in student writings, the precision rates differ across error types, and the identified errors constitute only a small proportion of all the errors present in the evaluated texts (Bai and Hu, 2017). AWE can effectively supplement but cannot supplant peer and instructor feedback activities in the EFL writing classroom, given the low precision and accuracy rates of the AWE program in identifying a range of errors (Bai and Hu, 2017; Yao et al., 2021). Directing attention to these mechanical errors can divert students’ attention to less important features of writing, thus biasing their attention to these errors and guiding their attention away from the content or structure of their essays (Graham and Santangelo, 2014). Therefore, learners in this study somewhat utilized the AWE program’s strengths in providing diagnostic feedback on the microstructural aspects of students’ writings (e.g., grammar, mechanics, and usage conventions), enabling the reviewers to be cognizant of problems in other aspects, like layout, content, and style and tone, which constitute the essential elements of a business letter.

Our findings partially corroborated Cho and Cho’s (2011) finding that providing praise comments about high-level writings was positively related to the providers’ quality of drafts. The immediate posttest (revised draft) and the delayed posttest in our study correlated significantly with the strength comments about high-quality writings. Though the strength comments given by our participants were specific and pertinent, our regression analyzes failed to observe that the strength comments about high-quality writings could significantly predict their own writing qualities at any of the posttests after the intervention. Lu and Zhang (2012) also did not find a significant relationship between praise and performance, and they attributed this finding to the broad and vague praise comments (e.g., “Good job.”). Another plausible explanation is that feedback affected learning and performance in different ways. Namely, feedback provided without explanations can improve performance, but not learning, because critical comments and directive comments are key to high-road transfer, as implied by Reiff and Bawarshi (2011). Thus, feedback should be provided with critical and constructive comments and explanations for learning to occur across different contexts.

## Conclusion

This study, grounded in the Vuogan and Li’s (2022) tripartite peer feedback framework and Cho and MacArthur’s (2011) *learning-by-reviewing* hypothesis, provided preliminary evidence that peer feedback together with weakness feedback comments for medium-level peers’ drafts contributed positively to learners’ genre awareness in business letter writing across the tests. First, peer feedback activities did enhance reviewers’ genre awareness not only in the immediate posttest, but also in the transfer test and the delayed transfer posttest. The significant improvement for the experimental group at the transfer posttest and the delayed transfer posttest compared with the control group suggested that peer feedback exerted positive impact on learners’ learning outcomes in business letter writing. Second, this study delineated how different types of feedback comments bore relationship with learners’ subsequent writing performance after the intervention. Providing weakness comments seemed to be more effective than offering strength comments in terms of the reviewers’ gains in their writing performance. Third, providing weakness comments about medium-quality drafts could effectively predict learners’ writing gains on the revised drafts, transfer task and delayed transfer task.

Based on the findings of our study, several pedagogical implications could be made in order to effectively employ peer feedback practice in the EFL business writing classroom.

Firstly, while previous studies inform teachers of the value of peer feedback on providers’ writing outcomes, this study suggests that well-organized peer feedback activities can be utilized to help learners achieve not only low-road transfer, for example, to automatize the genre conventions in business letter writing, but also high-road transfer, which requires reflective thought and the related ability to seek connections between contexts and to abstract and draw from prior skills and knowledge (Reiff and Bawarshi, 2011), like considering the style and tone in composing a business letter. Students should be encouraged to adopt an audience awareness approach to writing—to consider their audience and the complexities of their writing context carefully when composing texts. In this study, learners’ performance at the transfer task for the experimental group were noticeably better than the control group who did self-revising. Teachers need to create opportunities for learners to take an audience perspective when writing. As suggested by Noodine (2002), in addition to the instructor offering one or more model papers for students to emulate, students should be offered structured guidance for how to review papers and spend time reading and critiquing the writing of their peers.

Secondly, when scaffolding learners to give feedback to peers’ drafts, teachers can demonstrate to the class that the value of elaborating on weakness of drafts is noteworthy. As St John (1996) lamented, the materials that provide examples and models for learners to copy are far from enough to develop language awareness. Based on learners’ remembering and understanding genre features of the business letter writing, evaluating and critiquing peers’ drafts coupled with offering constructive suggestions may actively engage learners in the process of reviewing. On the basis of learners’ remembering and understanding genre features of the business letter writing, evaluating and critiquing peers’ drafts coupled with offering constructive suggestions may actively engage learners in the process of reviewing. Peer feedback activities can enable students to recognize the weakness of peers’ drafts, as well as to recognize those of their own drafts. The experience of evaluating others’ work and giving explanatory feedback enabled the reviewers to write higher quality essays (Philippakos and MacArthur, 2016, p. 430).

Finally, teachers should be encouraged to expose learners to multiple peer drafts during reviewing, especially drafts of medium-quality. In our study, the medium-quality drafts seem to be more effective materials than low-and high-quality texts to be used for learners to provide comments. It is hoped that peer feedback applied in the EFL business writing classroom can better prepare learners for their communicative competence in their future career and ease their transition into workplace writing.

Admittedly, the findings of the study are tentative and should be interpreted cautiously. Several limitations in the study merit further research. First, instructors in this study were not required to provide feedback to each learner. Future research may incorporate a comparison group who receive teacher feedback to better understand different effects of feedback on learners’ writing performance. Second, this study focused only on feedback providers’ learning gains, while in actual classroom settings, students take both roles as writer and reviewer to participate in reciprocal peer review practice. Therefore, it would be equally meaningful to further explore feedback receivers’ perceptions of different feedback comments and the uptake of these comments in their revised drafts and the performances in the transfer test. Third, our study did not consider individual differences like learners’ learning styles and the critical thinking ability. Future study may explore the roles of individual differences in providing peer feedback.

## Data availability statement

The raw data supporting the conclusions of this article will be made available by the authors, without undue reservation.

## Ethics statement

The studies involving human participants were reviewed and approved by the Ethic Committee of School of Foreign Languages, Tongji University. The patients/participants provided their written informed consent to participate in this study.

## Author contributions

HW and XY contributed to conception and design of the study and analyzed the data. HZ conducted the experiment. XY interpreted the data results, supervised the study, and revised the draft. HW wrote the first draft. All authors contributed to the article and approved the submitted version.

## Funding

This research was supported by the Ministry of Education of the People’s Republic of China, Grant Number DBA210298.

## Conflict of interest

The authors declare that the research was conducted in the absence of any commercial or financial relationships that could be construed as a potential conflict of interest.

## Publisher’s note

All claims expressed in this article are solely those of the authors and do not necessarily represent those of their affiliated organizations, or those of the publisher, the editors and the reviewers. Any product that may be evaluated in this article, or claim that may be made by its manufacturer, is not guaranteed or endorsed by the publisher.

## Footnotes

^1^Differences Between Academic and Business Writing. https://darkwing.uoregon.edu/~ddusseau/101/199/199differences.htm. Accessed on 7th October, 2022.

^2^ www.pigai.org
