# Supplementary material for: The effects of providing peer feedback on learners’ genre awareness in English as a foreign language business letter writing
Source: Front Psychol. 2022 Dec 1;13:1059555. doi: 10.3389/fpsyg.2022.1059555 (PMC9751833; doi:10.3389/fpsyg.2022.1059555)
Supplement: Supplementary file 1 [file Data_Sheet_1.docx]

# Appendix A. Writing Prompts

| **Task** | **Prompt title** | **Situation** | **Task** |
| --- | --- | --- | --- |
| 1 | A letter of invitation to market the new games | You work as Assistant Sales Manager for Cybergames Ltd, which designs original training games for business organizations. You are planning an exclusive half-day demonstration of your new games ‘Takeover’ and ‘Marketing Manager’. This will take place at the head office for special customers only. Your Chief Designer, Ms Pauline Davi, will open the demonstration. There will also be a buffet lunch. Your company is based at: 44 Regent Street, London WC1 2EE | Write a letter of invitation to Mr Roland Jenks, the Sales director for Business Lines Ltd who, you hope, will market the new games, include details such as date, times, venue, etc, plus a timetable for the half day. Give him a brief description of the new games.The address of Business Lines Ltd is: 2 Bridge Street, Oxford, OS2 4JF. You may invent any details that you feel are necessary. |
| 2 | A letter of looking to establish a new market | You work in the Sales Department of Starlight Perfumes Ltd which manufactures ladies’ perfumes. You are looking to establish a market in eastern Europe. Recently in London you met Mrs Liliana Vucic, who is Chief Buyer for a chain of department stores in Yugoslavia. Her contact address is: 64 Skadalijie, 11000 Belgrade. | Write a letter to Liliana Vucic from your offices at 4 Rosewood Square, Chelsea London SW4 6 BJ. Tell her about your products and ask for her advice on establishing a new market in eastern Europe. You may invent any details that you feel necessary. |
| 3 | A letter to a job candidate for an interview | You work as an administrative assistant at the Gunter Hopp Fitness and Leisure Club, situated at: Loniginstrasse 54, 11203 Berlin. Your manager is Herr Rudi Barenbohm and he has given you a list of candidates for a vacancy at the club. Interviews will take place on 16 November. Before the interviews, which start at 2:00pm, there will be a tour of the club and a meeting with other staff. These activities will take an hour, followed by the interview which will be in alphabetical order. | Write a letter to one of the job candidates, Ms Natasha Braun, giving details and timings for the afternoon. Her address is: Michelangelostrasse 19, 10409 Berlin. Rudi has asked you to remind all candidates to bring along their qualification certificates. You may invent any details that you feel are necessary. |

**Appendix B. Example Papers**

**The Example Letter of Low Quality**

5,2022

Dear Mr Roland Jenks

Hello, I am the assistant sales manager of Cybergames Ltd. Our company is planning an excusive half-day demonstration of two games which named "Takeover" and "Marketing Manager". The activity will be held on 5,28,2022 at the head office and for special customers only. Our Chief Designer Ms Pauline Davi will be the host of the demonstration. After that, there will be a buffet lunch which you can enjoy joyfully.

Because our games are still fresh and do not have many investors. We sincerely hope you can give us a hand by market the games. The games have passed all of our tests and are pretty good. What' more, they are useful to cultivate marketing talents. They absolutely worth your expectation. Hope you could have a try and help us to improve the quality by giving some suggestions. Our address is 44 Regent Street, Oxford, OS2 4JF. We are looking forward to welcome your attendance!

Yours Sincerely

Cybergames Ltd

**The Example Letter of Medium Quality**

May 25,2022

Business Lines Ltd

2  Bridge Street, Oxford, OS2 4JF.

Dear Mr Roland Jenks

I'm delighted to invite you to attend the buffet lunch that held at our company head office (44 Regent Street, London WC1 2EE ) at 12:00 on May.25th. This buffet lunch is only for special customers and is hosted by our chief designer, Ms. Pauline Davi, and will mainly introduce our new games "Takeover"and "Marketing Manager ".

After such a long time of cooperation, I believe you must have a certain understanding of our company.This buffet lunch mainly to introduce you to our new games. These two games are mainly developed for some business organizations, which can help their employees understand their work conditions, deal with various situations at work in advance and provide better training for employees. At that time, I will further introduce these two games. You can also have a more in-depth experience and give your suggestions. We'd like to see that after this buffet lunch, you can learn more about our new games and market them.

It may rain on May 25. You can take an umbrella in case. If you don't, we will prepare for you. The buffet lunch starts at 12 o'clock. You can arrive half an hour in advance. At that time, the staff will guide you and provide some help. 12: 30-13:30 is our official demonstration. After that, you can experience it yourself.

Thank you very much for reading this email in your busy schedule. We would appreciate it if you could come.

Sincerely yours

Cybergames Ltd

44 Regent Street, London WC1   2EE

Jane Assistant Sales Manager

**The Example Letter of High Quality**

Cybergames., Ltd.

44 Regent Street

London

WC12EE

Tel: 004-487-0036

18 May, 2022

Mr. Roland Jenks

Sales Department

Business Lines.,Ltd

2 Bridge Street

Oxford

OS24JF

Dear Mr. Jenks

We would like to invite you to attend a half-day demonstration of our new games--'Takeover' and 'Marketing Manager', which is to be held at 9:00 am at the head office of our company on May 30, 2022.

In view of the good cooperation experiences between you and us, we will launch original training games in this half-day demonstration and invite you to come. Both games are specially designed for senior employee training. 'Takeover' will help general manager make decisions when facing difficulties, while 'Marketing Manager' will teach you methods for success in positive team building, so you are kindly invited to help marketing the games. What's more, we strongly desire for carrying out follow-up cooperation with you in promoting the games. We firmly believe that you will benefit from the training of employees.

The following is a brief schedule of the demonstration:

- **9:00 am- 10:00 am：** An opening speech and a specific introduction of games by Game Chief Designer--Ms. Pauline Davi
- **10:00 am- 11:00 am：**Playing demo versions of games & Q&A session
- **11:00 am- 12:00 am：**A buffer lunch with free coffee
- **12:00 am- 13:00 pm：**Discussion for follow-up cooperation

We do hope you will be able to join us on the occasion, and look forward to meeting you at the demonstration.

Yours sincerely

Lily Sheng

Assistant Sales Manager

**Appendix C. Descriptors for scoring rubrics**

layout (0-5): The writer uses the correct and complete form for a business letter. A business letter contains the essential parts: heading, inside address, salutation, body, closing, and signature.

content (0-15): Each part is complete and correct. The body contains courteous, formal language and all the details that the audience will need.

style/Tone (0-4): It targets message to audience, acknowledges and meets audience needs (explicitly, if possible), and chooses words that are clear, descriptive, and accurate.

accuracy (0-6): There are few or no errors in mechanics, usage, grammar, or spelling. a half mark deducted for each error of spelling, grammar, and punctuation
